# Impact of Ascorbic Acid on Zero-Valent Iron Nanoparticle and UV-B Mediated Stress in the Cyanobacterium, *Fremyella diplosiphon*

**DOI:** 10.3390/microorganisms11051245

**Published:** 2023-05-09

**Authors:** LaDonna Wyatt, Samson Gichuki, Yavuz S. Yalcin, Viji Sitther

**Affiliations:** Department of Biology, Morgan State University, 1700 E. Cold Spring Lane, Baltimore, MD 21251, USA

**Keywords:** ascorbic acid, fatty acid methyl esters, lipids, nZVIs, *Fremyella diplosiphon*

## Abstract

*Fremyella diplosiphon* is an ideal third-generation biofuel source due to its ability to produce transesterified lipids. While nanofer 25s zero-valent iron nanoparticles (nZVIs) improve lipid production, an imbalance between reactive oxygen species (ROS) and cellular defense can be catastrophic to the organism. In the present study, the effect of ascorbic acid on nZVI and UV-induced stress in *F. diplosiphon* strain B481-SD was investigated, and lipid profiles in the combination regimen of nZVIs and ascorbic acid compared. Comparison of *F. diplosiphon* growth in BG11 media amended with 2, 4, 6, 8, and 10 mM ascorbic acid indicated 6 mM to be optimal for the growth of B481-SD. Further, growth in 6 mM ascorbic acid combined with 3.2 mg/L nZVIs was significantly higher when compared to the combination regimen of 12.8 and 51.2 mg/L of nZVIs and 6 mM ascorbic acid. The reversal effect of UV-B radiation for 30 min and 1 h indicated that ascorbic acid restored B481-SD growth. Transesterified lipids characterized by gas chromatography–mass spectrometry indicated C16 hexadecanoate to be the most abundant fatty acid methyl ester in the combination regimen of 6 mM ascorbic acid and 12.8 mg/L nZVI-treated *F. diplosiphon*. These findings were supported by microscopic observations in which cellular degradation was observed in B481-SD cells treated with 6 mM ascorbic acid and 12.8 mg/L nZVIs. Our results indicate that ascorbic acid counteracts the damaging effect of oxidative stress produced by nZVIs.

## 1. Introduction

From an ecological, economic, or evolutionary standpoint, cyanobacteria are the most predominant photosynthetic prokaryotes capable of utilizing solar energy to produce biomass precursors [1]. The ubiquitous nature of these organisms allows them to function in trophic energy dynamics of both aquatic and terrestrial ecosystems. Their ability to thrive in the presence of minimal nutrients and abiotic conditions such as carbon dioxide, mineral salts, and light allows their cultivation in marginal lands and minimizes competition with food crops such as corn or sugarcane [2]. Adaptive diversification and inclusive survival of cyanobacteria in a range of ecological niches have ensured these large and diverse arrays of photosynthetic microbes compete successfully on the planet. Additionally, cyanobacteria have attracted considerable attention as a sustainable source of energy due to their metabolic versatility. Several strategies such as genetic transformation to overexpress target genes in cyanobacteria have been pursued to enhance their biofuel production efficacy [3].

In recent years, cultivation of various algal and cyanobacterial strains with zero-valent iron nanoparticles (nZVIs) has been reported to boost their growth and lipid production [4,5]; however, high reactive oxygen species (ROS) accumulation in cells is inevitable and can be detrimental to the organism [6]. In addition, UV-B radiation can drastically affect cyanobacteria by inducing DNA-DNA crosslinking and breaks, base modifications and translocations, and ATM-mediated phosphorylation of the BH3-interacting domain death agonist protein [7]. In addition to damaging the photosynthetic apparatus resulting in reduced photosynthesis, UV-B is also known to induce amino acid modification and increased susceptibility to proteolysis and fragmentation of amino acids in these microorganisms [8].

Commonly known as vitamin C, ascorbic acid plays multiple functional roles such as antioxidant induction, reducing agent, stabilizer, and modifier of oxidative-reduction processes [9]. It has been reported that ascorbic acid regulates cell division and growth in cyanobacterial and algal cultures by modulating signal transduction [10]. In addition to its role as a major redox buffer and cofactor, ascorbic acid can also regenerate other antioxidants by scavenging toxic free radicals and other ROS produced during cell metabolism [11]. Excessive ROS produced at above-optimal levels can damage cellular components such as proteins, lipids, and DNA, which can impair metabolism resulting in membrane damage and ultimately lead to cell death [12]. In cyanobacteria, ROS are generated in abundance by the photosynthetic process at the expense of their efficiency, and specific gene expression profiles are triggered by over excitation of photosystem II [13]. Considering the necessity to perceive fluctuations in factors to regulate these protective mechanisms, the photosynthetic apparatus is a major sensor of environmental and metabolic changes, and transmits retrograde signals to the nucleus for changes in gene expression.

The reversal of ROS in cyanobacteria is a promising cellular survival mechanism to increase the efficiency of biofuel production, and has great potential in the development of sustainable energy solutions. Of the various cyanobacterial strains, *Fremyella diplosiphon* is a widely studied model organism known for its adaptive growth, capability in varying light intensities, and survival in varying environmental regimes. Additionally, its ability to produce lipids and desirable essential fatty acid methyl esters (FAMEs) makes it an ideal third-generation microbiological biofuel factory. To our knowledge, there are no reports on the impact of ascorbic acid and reversal of nZVI and UV-mediated ROS in *F. diplosiphon*. The present study was aimed to investigate the impact of ascorbic acid on nZVI and UV-induced stress in *F. diplosiphon* strain B481-SD. In addition, total lipids in nZVIs and the combination regimen of nZVIs and ascorbic acid-treated *F. diplosiphon* were transesterified to FAMEs and compared to the control.

## 2. Materials and Methods

### 2.1. Strain and Experimental Conditions

The strain B481-SD, a wildtype B481 *F. diplosiphon* strain engineered with the sterol desaturase gene (accession MH329183), was used in this study. All cultures were grown in liquid BG-11 medium containing 20 mM HEPES (BG-11/HEPES) under continuous shaking at 170 rpm, 28 °C, and ambient CO_2_ in an Innova 44R incubator shaker (Eppendorf, Hamburg, Germany). Light intensity in the shaker was adjusted to 30 μmol/m^2^/s using a LI-190SA quantum sensor (Li-Cor, Lincoln, NE, USA). Nanofer 25s nZVIs with an average size of 50 nm and surface area 20–25 m^2^/g (Nano Iron, Rajhrad, Czech Republic) were used in this study.

### 2.2. Effect of Ascorbic Acid on the Reversal of Zero-Valent Nanoparticle and UV-Mediated Stress in F. diplosiphon

The impact of 2, 4, 6, 8, and 10 mM ascorbic acid (L-ascorbate, MilliporeSigma, Burlington, MA, USA) on the growth of *F. diplosiphon* B481-SD was tested to determine the optimal concentration. In addition, the impact of ascorbic acid at 6 mM was tested in the reversal at 3.2, 12.8, and 51.2 mg/L nZVI-mediated stress. Cells that were grown in the absence of ascorbic acid served as the control. In addition, the impact of ascorbic acid on the reversal of UV-B stress in *F. diplosiphon* was investigated by exposing B481-SD cells to simulated UV-B conditions (Omaykey UV-B lamp). Cultures were grown to an optical density of 0.1 (OD_750_) in 250 mL flasks and exposed to UV-B at time intervals of 30 min and 1 h each day for 15 days to simulate the sun’s UV-B radiation effects. Cultures that were not treated with UV-B radiation served as control. Growth as a measure of optical density was determined every three days for a period of 15 days. Cultures were grown under constant shaking at 28 °C and 170 rpm, with an initial optical density of 0.1 at 750 nm. Three replicates were maintained and the experiment repeated once.

### 2.3. Microscopic Observations of F. diplosiphon Treated with Zero-Valent Iron Nanoparticles and Ascorbic Acid

At day 15 of treatment, *F. diplosiphon* B481-SD filaments in the control and nZVI-treated cultures were observed using a Bio-Tek microscope with Lionheart FX, and images captured in a manual mode at 20× in the GFP channel. To assess the membrane integrity, the SYTO 9 Bacterial Viability kit (Molecular Probes, Invitrogen, UK) was used. A 50-μL aliquot of each sample was diluted in 0.95 mL of dechlorinated filtered tap water and stained with SYTO 9. A 3 μL volume of an equal proportion of SYTO 9 was added to the sample and incubated in the dark at room temperature (20 °C) for 15 min, followed by filtration through a black polycarbonate Nucleopore^®^ membrane (0.2 μm pore size; Whatman, UK). Subsequently, the membranes were air dried and mounted onto glass slides with non-fluorescence immersion oil and a cover slip.

### 2.4. Total Lipid Extraction in F. diplosiphon

*F. diplosiphon* B481-SD cultures were grown in BG11/HEPES medium amended with 3.2, 12.8 and 51.2 mg/L nZVIs and ascorbic acid at 2, 4, 6, 8, and 10 mM. Cultures were grown under continuous shaking at 170 rpm and 28 °C for a period of 15 days. Cells were centrifuged using a Avanti-J25I with a JA25.50 rotor (Beckman-Coulter, Pasadena, CA, USA), lyophilized overnight, and sonicated in 5 mL chloroform:methanol (2:1) for 30 s. Total lipids were extracted using a 2:1 chloroform: methanol mixture according to the method of Folch et al. [14]. The mixture was agitated for 15–20 min in an orbital shaker after dispersion at room temperature and the homogenate centrifuged to recover the liquid phase. The solvent was washed with 0.2 volumes (1 for 5 mL) distilled H2O, vortexed briefly, and centrifuged at 2000 rpm to separate the phases. The lower phase was transferred to a pre-weighed vial and the interface was rinsed twice using methanol:water (1:1) without mixing the whole preparation. The lower chloroform phase containing lipids was evaporated under vacuum in a rotary evaporator after centrifugation and siphoning, and the extracted lipids were used for transesterification [15]. Three replicates were maintained and the experiment repeated once.

### 2.5. Transesterification of F. diplosiphon Extracted Lipids

The impact of ascorbic acid and nZVIs on transesterified lipids was investigated by growing B481-SD cultures at 3.2 and 12.8 mg/L nZVIs and 6 mM ascorbic acid under conditions mentioned in Section 2.1. After 15 days, cultures were centrifuged at 7300 rpm and the biomass lyophilized. Lipids in *F. diplosiphon* grown in the various treatments were extracted using a one-step transesterification procedure for in situ biofuel production [16]. Extracted lipids were transesterified by reacting 100 mg lyophilized cells with acidified methanol in a CEM multimode microwave, resulting in simultaneous cell lysis and transesterification of fatty acids. Briefly, 2 mL methanol containing 1.8% sulfuric acid (*v*/*v*) was added to the reaction vessel containing the lyophilized cells and a PTFE-coated stir bar (50 mm), and the microwave set to 80 °C, 30 s ramp time, 20 min hold time, and 500 W or 25 W per sample. Reactions were stopped by the addition of 5 mL chloroform to the reaction vessel. Phase separation was accomplished by adding the methanol–chloroform solution to 5 mL water followed by centrifugation at 2000 rpm. The methanol and sulfuric acid were partitioned with water in the upper phase, while fatty acid methyl esters with chloroform in the lower organic phase.

### 2.6. Gas Chromatography-Mass Spectrometry Analysis of Transesterified Lipids

Fatty acid methyl esters in *F. diplosiphon* transesterified lipids were subjected to GCxMS using a Shimadzu QP2010SE GC-MS with AOCi+s autosampler at the Johns Hopkins University (Baltimore, MD) mass spectrometry facility. Three biological replicates of each sample were analyzed. Split GC injection at the ratio of 10:1 with column DB5-ms, 30 m long, 0.25 mm ID, 0.25 um film thickness, and 10 m length guard column was used. Transesterified products were dissolved in acetone, and 1 μL sample was injected into the instrument using an autosampler. The injector temperature was maintained at 300 °C and transfer line interface at 300 °C, with the source temperature at 200 °C. The oven temperature was ramped from 130 °C (10 min) to 160 °C (7 min), 160 °C to 190 °C (7 min), 190 °C to 220 °C (22 min), and 220 °C to 250 °C (17 min) at a rate of 10 °C min^−1^ for each step. Positive ionization mode with a maximum scan range from 45–600 @ 1250 Da/sec without selected ion monitoring was used. Solvent cut time was 3.5 min with ionizing electron energy 70 eV and gas flow 1 mL/min of Helium. Acetone was used as a solvent blank and ethyl ethanoate as equipment blank, and kept constant throughout all experiments. We identified the peaks by comparing mass spectra to the lipid Web Archive of FAME mass spectra at https://www.lipidmaps.org/resources/lipidweb/index.php?page=ms/methesters/me-arch/index.htm (accessed on 8 April 2021). The tandem match of identified FAME peaks was determined by comparing mass spectra (M) and mass-to-charge (*m*/*z*) ratio from the lipid web’.

### 2.7. Statistical Analysis

Growth, total lipids, and FAME content were reported as a cumulative treatment mean ± standard error. Statistical significance was determined using one-way analysis of variance and Tukey’s honest significant difference post-hoc test at 95% confidence intervals (*p* < 0.05). The single-factor, fixed-effect ANOVA model, Yij = μ + αSi + εij, was used where Y is the total lipid content in strain i and biological replicate j. The μ represents overall total lipid content with adjustments from the effects of strain (αS), and εij is the experimental error from strain i and biological replicate j.

## 3. Results

### 3.1. Impact of Ascorbic Acid on F. diplosiphon Growth

**Figure 1:**
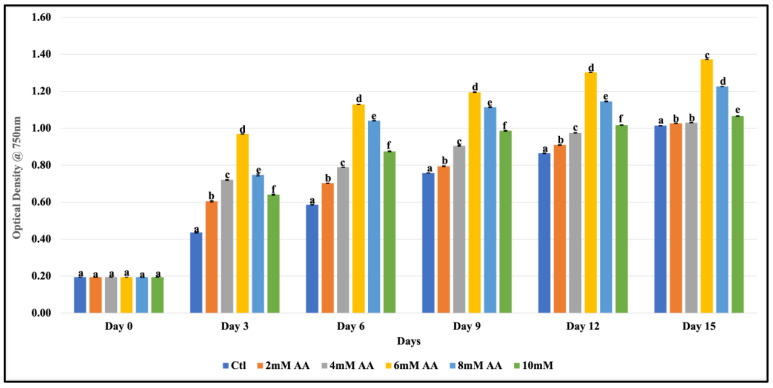
Growth of *Fremyella diplosiphon* strain B481-SD in BG11/HEPES medium containing 2, 4, 6, 8, and 10 mM of ascorbic acid (AA) over a period of 15 days. Different letters above the error bars indicate significant difference (*p* < 0.05) among treatment means (Tukey’s post-hoc test). Error bars indicate standard error (SE) of the mean.

We tested the growth of *F. diplosiphon* B481-SD in varying concentrations of ascorbic acid from 2 to 10 mM to determine the optimal requirement. Our studies indicated that ascorbic acid at all concentrations (2, 4, 6, 8, and 10 mM) significantly increased growth when compared to the untreated control (Figure 1). This increase was observed at all intervals tested, from day 3 to day 15. In addition, we observed 6 mM ascorbic acid to significantly enhance growth (OD_750_ nm) when compared to 2, 4, 8, and 10 mM ascorbic acid through the 15-day testing period (*p* < 00.5).

### 3.2. Ascorbic Acid-Mediated Growth in F. diplosiphon Exposed to Varying Zero-Valent Iron Nanoparticle Concentrations and UV-B Radiation

**Figure 2:**
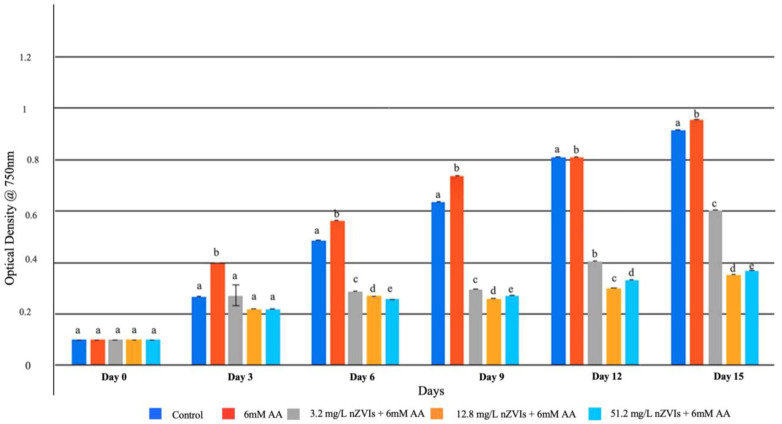
Growth of *Fremyella diplosiphon* strain B481-SD exposed to 3.2, 12.8, and 51.2 mg/L zerovalent nanofer 25s nanoparticles (nZVIs) and 6 mM ascorbic acid (AA) over a 15-day period. Average optical density at 750 nm in three biological replicates is shown for each time point. Different letters above the error bars indicate significant difference (*p* < 0.05) among treatment means (Tukey’s post-hoc test). Error bars indicate standard error (SE) of the mean.

The effect of ascorbic acid in counteracting nZVI-mediated oxidative stress was studied by exposing B481-SD cells to a combination treatment of 6 mM ascorbic acid and nZVIs at 3.2, 12.8, and 51.2 mg/L. Of the concentrations tested, significant enhancement of growth (*p* < 0.05) was observed in 6 mM ascorbic acid on days 3, 6, 9 and 15, compared to the untreated control, and the combination regimen of 3.2, 12.8, and 51.2 nZVIs as well as ascorbic acid (Figure 2). However, we did not observe any differences between the control and 6 mM ascorbic acid-treated cells on day 12 (*p* > 0.05). In addition, the combination regimen of 3.2 mg/L and 6 mM ascorbic acid indicated significantly higher growth when compared to the combination regimen of 12.8 and 51.2 mg/L nZVIs and 6 mM ascorbic acid (Figure 2). Cultures grown in the presence of ascorbic acid alone showed significantly higher growth compared to the untreated control and all combination regimes of ascorbic acid and nZVIs.

In order to study the impact of UV stress ascorbic acid, we exposed B481-SD to UV-B radiation for 30 min and 1 h and examined the reversal of ROS. We observed B481-SD treated with 10 mM ascorbic and 1 h UV-B exposure to show significant recovery compared to the cultures without ascorbic acid and 1 h UV-B exposure (*p* < 0.05). Similarly, B481-SD exposed to 30 min of UV-B and 10 mM ascorbic acid showed significant recovery compared to cultures grown in the absence of ascorbic acid and 30 min UV-B exposure (*p* < 0.05).

**Figure 3:**
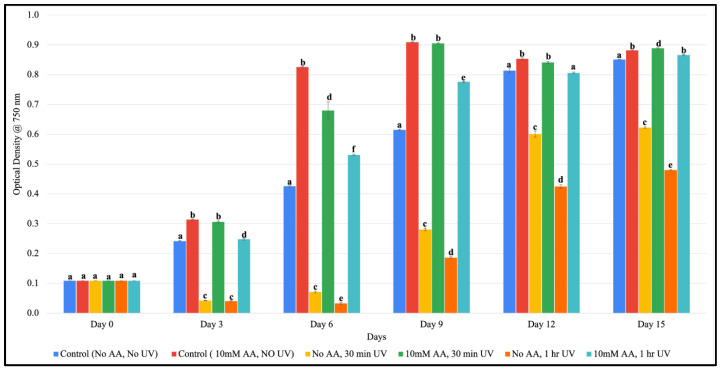
Growth of *Fremyella diplosiphon* B481-SD treated with zero-valent iron nanoparticles (nZVIs) and 10 mM ascorbic acid (AA) over a 15-day period. Average optical density at 750 nm in three biological replicates is shown. Reversal of UV-induced oxidative stress was observed on day 9 and continued until day 15. Different letters above the error bars indicate significant difference (*p* < 0.05) among treatment means (Tukey’s post-hoc test). Error bars indicate standard error (SE) of the mean.

Growth of B481-SD treated with ascorbic acid at 10 mM showed significantly higher OD_750_ values than cultures without ascorbic acid regimen at all intervals tested (Figure 3). Cultures exposed to UV-B for 30 min and 1 h started recovering on days 3 and 6, respectively. Additionally, cultures that were not treated with ascorbic acid following UV-B radiation lost pigmentation after UV-B exposure at both 30 min and 1 h intervals, and did not fully recover for the entire duration of the study. In addition, we observed notable bleaching of cells in cultures exposed to UV-B at both these intervals (Figure S1).

### 3.3. Microscopic Analysis of F. diplosiphon Treated with Ascorbic Acid and Varying Zero-Valent Iron Nanoparticle Concentrations

**Figure 4:**
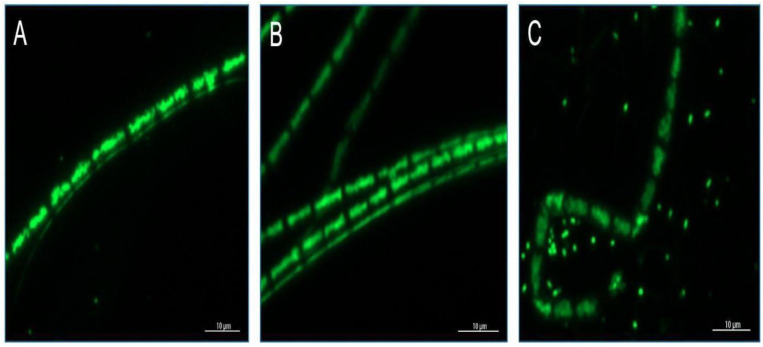
SYTO 9 labeled *Fremyella diplosiphon* treated with zero-valent iron nanoparticles (nZVIs) and ascorbic acid visualized using Bio-Tek microscope (**A**) untreated control, (**B**) 6 mM ascorbic acid, and (**C**) 6 mM ascorbic acid + 12.8 mg/L nZVIs.

The green-fluorescent nucleic acid stain SYTO-9 was used to visualize cellular viability and degradation after exposure of cells treated with nZVIs and ascorbic acid. Microscopic observations revealed cellular death in the combination regimen of 12.8 mg/L nZVIs and 6 mM ascorbic acid (Figure 4C). Cellular contours were intact at 6 mM ascorbic acid, indicating that the cells were not damaged compared to the combination regimen of 6 mM ascorbic acid and 12.8 mg/L nZVIs.

### 3.4. FAME Identification in F. diplosiphon Treated with Varying Zero-Valent Iron Nanoparticle Concentrations and Ascorbic Acid

We used one-dimensional gas chromatography to identify the FAMEs present in *F. diplosiphon* grown in individual treatments of 3.2 and 12.8 mg/L nZVIs, and the combination regimen of 6 mM ascorbic acid along with these nZVI concentrations. The FAME composition of *F. diplosiphon* based on lipid extraction and esterification of fatty acids from lyophilized biomass subjected to GC-MS is shown in Table 1. Our results showed that B481-SD cultures treated with 3.2, 12.8 mg/L nZVIs, and the combination regimen of 3.2, 12.8 mg/L nZVIs, and 6 mM ascorbic acid contained higher hexadecanoate (16:0) FAMEs compared to the untreated control. A sample chromatogram of hexadecanoate, which was identified as the primary fatty acid methyl ester in *F. diplosiphon* transesterified lipids, is shown (Figure S2). Other important FAMEs identified in the combination regimen included methyl esters of monoenoic fatty acids, hydroxy fatty acids, saturated straight chain fatty acids, trienoic acids (including MTAD adducts), hydroxy fatty acids, and natural fatty acids (methyl ferulate) (Table 1). Interestingly, we observed more FAMEs in B481-SD treated with 3.2 mg/L nZVIs, compared to all other treatments.

## 4. Discussion

In recent years, the use of nanoparticles has gained tremendous interest due to their potential to improve air, water, and soil quality. Specifically, metallic nanoparticles are used as catalysts to enhance biodiesel production due to their higher surface area/volume ratio, reactivity, and light-scattering properties at optimal concentrations [5,17]. These nanomaterials are also known to enhance biohydrogen, biogas, and bioethanol production, thus increasing the energy-conversion efficiency of microorganisms [18]. 

Of the various nanomaterials, nZVIs have unique applications since they contain zero-valent iron molecules that can be converted to water-soluble Fe^+2^, functioning iron-dependent enzymes [19]. As a micronutrient, iron is essential for the metabolism and growth of cyanobacteria and functions as a cofactor in enhancing nitrogenase activity, a crucial enzyme responsible for nitrogen fixation in cyanobacteria [20]. In addition to nanomaterials, optimal UV-B radiation is essential for energy-dependent processes such as photosynthesis, growth, survival, cell differentiation, genome integrity, and total lipid profiles [3,21]. Nevertheless, cyanobacteria are challenged by toxic ROS due to drastic changes in metal availabilities and intense UV-B illumination, when the amount of electrons they produce exceeds the amount they need to assimilate inorganic nutrients. It is therefore crucial to analyze the effects of metallic and UV-B mediated stress in these ancient organisms. 

In our study, the individual effect of ascorbic acid ranging from 2 to 10 mM was tested, and 6 mM ascorbic acid was found to significantly enhance the growth of strain B481-SD (Figure 1). It should be noted that *F. diplosiphon* strains can possess varying levels of tolerance to chemicals. A study by Yalcin et al. [22] reported contrasting characteristics in the tolerance of *F. diplosiphon* strains (B481-SD and B481-WT) to different classes of antibiotics. It is possible that overexpression of the sterol desaturase gene in B481-SD could have enhanced other genes as well, thus augmenting its tolerance to ascorbic acid. A study by Bhatia et al. [23] reported 6–10 mg/L ascorbic acid to significantly increase the growth rate and biomass production in *Spirulina platensis*, in addition to chlorophyll *a*, phycocyanin, carotenoids, and protein content. In addition, the frequency of spirally coiled filaments was increased to 41% compared to the control at 8%.

In further studies on the reversal of oxidative stress, we observed that 6 mM ascorbic acid when coupled with 3.2 mg/L nZVIs significantly increased *F. diplosiphon* growth, compared to the combination regimen of 6 mM with 12.8 and 51.2 mg/L nZVIs on days 6, 9, 12 and 15 (Figure 2). As reported by in an earlier study, nZVI concentrations above 12.8 mg/L resulted in significantly high oxidative stress; however, cultures exposed to nZVIs at optimal levels increased cell growth and lipid production [5]. It is known that higher concentrations of nZVIs have the capacity to react with oxygen, resulting in the rapid generation of free radicals due to their redox properties [24]. As a potent antioxidant, ascorbic acid provides first line defense against UV radiation and can scavenge ROS and protect cells from oxidative damage, in addition to enhancing growth rate by stimulating cell expansion and solute uptake [25]. Additionally, ROS can be neutralized by enzymatic activities such as superoxide dismutase, catalase, or glutathione reductase by donating electrons, thus reducing their reactivity [26]. Thus, cellular macromolecules such as lipids, proteins, and DNA are protected from mutations and other genetic damages. Disruption of cellular organelles and physiological processes due to excessive ROS in cyanobacteria is a well-known fact [27]. For example, a study has shown that nanoparticles can be toxic to cyanobacteria by increasing ROS activity, thus damaging photosynthetic activity, cellular pigmentation, and growth [28]. Thus, it is possible that ascorbic acid can restore cyanobacterial vital cellular processes with its antioxidant activity. Microscopic analysis using SYTO 9 supported our hypothesis, where we observed stained nuclei and cytoplasmic debris. As a fluorescent nucleic acid stain widely used in fluorescence microscopy, this assay supported our observations, indicating cell disruption in treated samples.

Although solar ultraviolet radiation is crucial for optimal cellular growth, the degree of UV-B radiation is another major limiting factor in the large-scale cultivation of cyanobacterial biofuel production. As revealed in our study, ascorbic acid at 10 mM protected *F. diplosiphon* from UV-B radiation at 30 min and 1 h exposure and was not lethal at all intervals tested (Figure 3). Cultures that were not treated with ascorbic acid following UV-B radiation at both 30 min and 1 h intervals did not recover in the entire duration of the study (Figure S1), indicating irreversible cellular damage. UV-B radiation can disrupt the cyanobacterial photosynthetic apparatus by damaging pigments such as chlorophyll and phycobilins, photosystems, and the electron transport chain [29]. This can result in the production of ROS and the accumulation of oxidized products, which can cause further damage to the cells. Results of our study reveal that ascorbic acid protects *F. diplosiphon* from UV-related damage. It is possible that DNA repair mechanisms are promoted, thereby enhancing photosynthesis and ultimately cyanobacterial energy production. In a study by He and Hader [30], the presence of ascorbic acid and *N*-acetyl-l-cysteine reversed oxidative stress and protected *Anabaena* sp. from chlorophyll bleaching and UV-B, resulting in a significantly higher survival rate. In addition, it is possible that ascorbic acid can facilitate the modulation of gene expression, thereby enhancing stress tolerance. The interactive effects of mannitol and ascorbic acid on the marine algae *Schizochytrium* sp. showed enhanced saturated and polyunsaturated fatty acids with a yield of 103.7% and 49.6% increment, respectively. Transcriptome changes investigated by RNA-sequencing revealed differential expression of lipid biosynthetic genes, in addition to transcripts encoding enzymes that catalyze the biosynthesis of steroids and carotenoids [31]. The addition of exogenous antioxidants resulted in the up-regulation of catalase, a key antioxidant enzyme that mediates intracellular ROS scavenging. 

Defense strategies such as the presence of an exopolysaccharide layer, carboxysomes to cater low carbon availability, antioxidant enzymes and non-enzymatic molecules such as tocopherol, ascorbic acid, and carotenoids enable cyanobacteria to thrive in extreme habitat [32]. In addition, these organisms accumulate lipids under stress conditions such as nutrient deprivation, exposure to UV-B, and salinity [33]. Since fatty acids are the building blocks of lipids, changes in the fatty acid composition can affect the fluidity and stability of the membrane. It is known that cyanobacterial lipids are modulated by ascorbic acid in a number of ways. A study on the effect of ascorbic acid on lipid metabolism and lipid peroxidation in *Microcystis aeruginosa* reported increased levels of unsaturated fatty acids and reduced lipid peroxidation, suggesting that ascorbic acid may protect cyanobacterial lipids from oxidative damage by stabilizing the double bonds in unsaturated fatty acids [34]. It is possible that ascorbic acid can up-regulate the expression of genes involved in fatty acid synthesis, leading to increased levels of unsaturated fatty acids in the membrane. An increase in the accumulation of saturated and unsaturated fatty acids in algae exposed to antioxidants was reported [31]. Analysis of differential genes involved in the process of lipid biosynthesis revealed up-regulation of two transcripts of the ketoacyl-reductase gene involved in the biosynthesis of fatty acids, two transcripts encoding diacylglycerol pyrophosphate phosphatase 1, and one transcript encoding glycerol-3-phosphate O-acyltransferase. 

In our study, we observed hexadecanoate as a primary fatty acid methyl ester, accounting for the bulk percentage in all nZVI-treated cultures exposed to ascorbic acid (Table 1). Identification of hexadecanoate as a vital component in cultures indicated that ascorbic acid as a reversal agent did not affect its biofuel potential. In a study by Gheda and Ismail [35], pronounced ratios recording 72.763% 11-octadecenoic acid methyl ester (38.344%), 9,12-octadecadienoic acid (Z,Z) methyl ester (22.771%), and hexadecanoic acid methyl ester were reported in *Oscillatoria amphigranulata*. In addition, McCracken et al. [36] observed that the anti-algal effects of unsaturated linkages of fatty acids extracted from *Chlamydomonas* were proportional to the number of unsaturated linkages. In our study, we observed that B481-SD treated with the combination regimen of 3.2, 12.8 mg/L nZVIs and 10mM ascorbic acid produced less saturated and polyunsaturated fatty acids compared to individual 3.2, 12.8 mg/L nZVIs. We hypothesize that ascorbic acid could decrease the number of saturated and polyunsaturated fatty acids and increase monosaturated fatty acids. A study by Chien et al. [37] revealed significant increases in monounsaturated fatty acids in 80 ppm to 200 ppm ascorbic acid when compared to untreated control. A number of factors such as the length of the carbon chain, the number of unsaturated linkages, and the position of double bonds can affect the anti-cyanobacterial activity of fatty acids as well.

## 5. Conclusions 

For the first time, we report the effect of ascorbic acid in the reversal of ROS produced by nZVI and UV-mediated stress in *F. diplosiphon*. Our results revealed that the organism uses ascorbic acid as a defense mechanism by protecting cellular components from nZVI-mediated oxidative damage. Comparison on FAMEs in transesterified lipids determined by GCxMS indicate the potential of the combination regimen of nZVIs and ascorbic acid as an efficient alternative to produce biofuels, due to the high proportion of FAMEs. In summary, supplementing ascorbic acid in *F. diplosiphon* B481–SD can help protect cells from the harmful effects of ROS-induced oxidative stress. Future investigations will aim to study lipid biosynthesis genes such as acetyl-CoA carboxylase and glycerol-3-phosphate acyltransferase in nZVI and ascorbic-acid-treated *F. diplosiphon* to understand defense mechanisms and signaling pathways that offer tolerance to these stressors. 

## Figures and Tables

**Table 1:** Fatty acid methyl esters in *Fremyella diplosiphon* treated with 3.2 and 12.8 mg/L zero-valent iron nanoparticles (nZVIs), and combination regimen of 3.2, 12.8 mg/L nZVIs, and 6 mM ascorbic acid (AA).

Treatment	Percentage Area	Group Name	Identity
Control	61.86	Methyl esters of saturated straight *chain fatty* acids (including those labelled with stable isotopes)	Hexadecanoate (16:0)
	23.12	Methyl esters of trienoic acids including (MTAD adducts)	Methyl 3c,9c,12c-octadecatrienoate (3,9,12 -18:3_
	6.95	Methyl esters monoenoic fatty acids (including dimethyl disulfide adducts)	Methyl 9-tetradecenoate
	2.14	Methyl esters of allenic acids	Methyl 4,5-trdecadienoate (4,5-13:2)
	1.98	Methyl esters of monoenoic fatty acids (including dimethyl disulfide adducts)	Methyl 9-tetradecenoate (9-14:1
3.2 mg/L nZVIs	70.85	Methyl esters of saturated straight chain fatty acids (including those labelled with stable isotopes)	Hexadecanoate (16:0
	20.23	methyl esters of tetra-, penta-, and hexaenoic fatty acids	methyl 5,8,11,14-octadecatetraenoate (18:4(n-4))
	3.88	methyl esters of acetylenic fatty acids	methyl octadeca-9-yn, trans-11-enoate
	1.38	Methyl esters of trienoic acids including (MTAD adducts)	methyl 8,11,14-heptadecatrienoate
	0.92	Methyl esters of natural cyclic fatty acids	Methyl 11-cyclopentylundecanoate
	0.92	Methyl esters of hydroxy fatty acids	Methyl 17-hydroxy-octadecanoate
	0.57	Methyl esters of trienoic acids including (MTAD adducts)	methyl 8,11, 14-heptadecatrienoate
	0.55	Methyl esters of trienoic acids including (MTAD adducts)	methyl 8,11, 14-heptadecatrienoate
12.8 mg/L nZVIs	70.64	Methyl esters of saturated straight chain fatty acids (including those labelled with stable isotopes)	Hexadecanoate (16:0)
	22.16	Methyl esters if trienoic acids including (MTAD adducts)	Methyl 3c,9c,12c-octadecatrienoate (3,9,12 -18:3_
	2.06	Methyl esters if trienoic acids including (MTAD adducts)	Methyl 3c,9c,12c-octadecatrienoate (3,9,12 -18:3_
	1.88	Methyl esters of trienoic acids including (MTAD adducts)	Methyl 8,11,14-heptadecatrienoate
	1.08	Methyl esters if monoenoic fatty acids (including dimethyl disulfide adducts)	methyl trans-3-hexadecenoate (3t-16:1)
3.2 mg/L nZVIs + 6 mM AA	62.9	Methyl esters of saturated straight chain fatty acids (including those labelled with stable isotopes)	Hexadecanoate (16:0)
	31.2	Methyl esters of monoenoic fatty acids (including dimethyl disulfide adducts)	Methyl trans-2-octadecenoate (2-18:1)
	2.63	Methyl esters of hydroxy fatty acids	Methyl 13-hydroxy-hexadecanoate trimethylsilyl ether derivative
12.8 mg/L + nZVIs 6 mM AA	72.48	Methyl esters of saturated straight chain fatty acids (including those labelled with stable isotopes)	Hexadecanoate (16:0)
	25.77	Methyl esters of saturated straight chain fatty acids (including those labelled with stable isotopes)	Methyl docosanoate (22:0)
	0.79	Methyl esters of trienoic acids including (MTAD adducts)	Methyl 8,11,14-heptadecatrienoate
	0.52	Methyl esters of hydroxy fatty acids	Dimethyl 9,10-dihydroxy-1,18-octadecanedioate TMS ether derivative
	0.45	Methyl esters of natural cyclic fatty acids	Methyl ferulate

## Data Availability

Data is contained within the article or Supplementary Materials.

## Supplementary Materials

The following supporting information can be downloaded at: https://www.mdpi.com/article/10.3390/microorganisms11051245/s1.
