# Supplementary material for: Impact of Ascorbic Acid on Zero-Valent Iron Nanoparticle and UV-B Mediated Stress in the Cyanobacterium, Fremyella diplosiphon
Source: Microorganisms. 2023 May 9;11(5):1245. doi: 10.3390/microorganisms11051245 (PMC10223273; doi:10.3390/microorganisms11051245)
Supplement: Supplementary file 1 [file microorganisms-11-01245-s001.zip › microorganisms-2313527-supplementary.pdf]

Supplementary figures

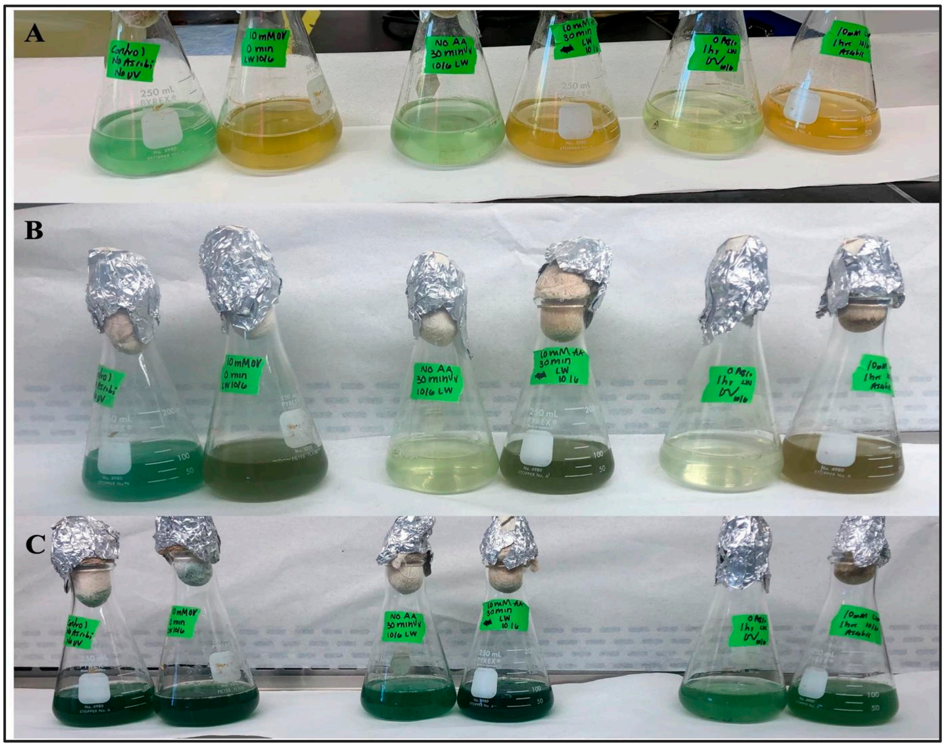

Figure S1. *Fremyella diplosiphon* cultures were treated with zero-valent iron nanoparticles (nZVIs) and 10 mM ascorbic acid on (A) day 3, (B) day 6, and (C) day 9. Reversal of UV-induced oxidative was observed day 9 and continued until day 15 (all images are not shown).

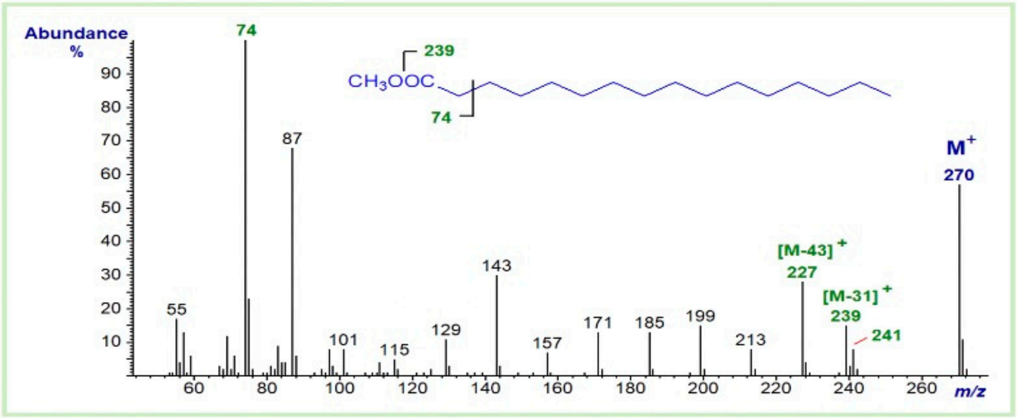

Figure S2. Representative chromatogram of hexadecanoate, which was identified as the primary fatty acid methyl ester identified in *Fremyella diplosiphon* transesterified lipids.
